# The Use of Accelerometers and Gyroscopes to Estimate Hip and Knee Angles on Gait Analysis

**DOI:** 10.3390/s140508430

**Published:** 2014-05-13

**Authors:** Francesco Alonge, Elisa Cucco, Filippo D'Ippolito, Alessio Pulizzotto

**Affiliations:** Dipartimento di Energia, Ingegneria dell'Informazione, e Modelli Matematici (DEIM), University of Palermo, Viale delle Scienze, Palermo, Italy; E-Mails: francesco.alonge@unipa.it (F.A.); elisa.cucco@unipa.it (E.C.); pulizzotto.alessio@gmail.com (A.P.)

**Keywords:** gait analysis, joint angles estimation, inertial sensors

## Abstract

In this paper the performance of a sensor system, which has been developed to estimate hip and knee angles and the beginning of the gait phase, have been investigated. The sensor system consists of accelerometers and gyroscopes. A new algorithm was developed in order to avoid the error accumulation due to the gyroscopes drift and vibrations due to the ground contact at the beginning of the stance phase. The proposed algorithm have been tested and compared to some existing algorithms on over-ground walking trials with a commercial device for assisted gait. The results have shown the good accuracy of the angles estimation, also in high angle rate movement.

## 1. Introduction

Gait analysis can be very important to support the rehabilitation of patients with a motor impairment and the monitoring of the patient's healing progress. Indeed, gait analysis can provide a quantitative description of the gait cycle and then improve the standard observational analysis. In the rehabilitation of motor function, the physiotherapist normally evaluates the improvement of the patient and his motor learning just by the visual information of the movements or by measurement of the time for a task; on the other hand, quantitative evaluation of movements, with a measurement system, are commonly used in research works. Gait analysis has attracted an increasing amount of attention from researchers and clinicians since the 1970s. Using video cameras, gait analysis based on highly accurate computer-based force plates was established in the 1980s and was applied in specialized motion laboratories. An optical 3D motion analysis system with a camera was used in [1,2] to detect walking motion; gait analysis with these methods is expected to really improve rehabilitation training, since they are highly accurate; however, they are expensive, need sophisticated instrumentation and specialized personnel and can be used in a laboratory or a clinical environment; therefore, they are difficult to apply in daily life applications. However, this standard gait analysis is possible on specialized laboratories with expensive equipment and lengthy set-up and post-processing times. Moreover, limitations in terms of moving area and gait cycles have been observed for patients.

In order to mitigate these problems, alternative gait analysis methods based on wearable sensors were studied and have shown great perspectives in the past two decades. Indeed, the term wearable implies that such a system is portable, lightweight and safe. In order for such a device to be accessible for home use, additional requirements are that the wearable sensor systems have to be cheaper and easy to operate.

In recent years, wearable sensors, such as accelerometers and gyroscopes, have been used in the measurement of human motion and in gait analysis; these inertial sensors have the properties of lower cost, small size robustness, easiness of setting and a number of efficient algorithms that exists, providing the estimation of gait parameters. Therefore, they are suitable for clinical application. Placed on foot segments, inertial sensors can be used for gait parameter estimation [3,4]. However, by adding sensors on leg segments, more information, such as joint angles, can be obtained. Anyway, since the inertial sensors are known for their offset, which results in accumulated drift after numerical operation, such as integration, several methods have been developed and described in the literature trying to minimize or solve this problem. Morris *et al.* [5] ideied the beginning and the end of the walking cycle and made the signals at the beginning and at the end of the cycle equal. Sabatini *et al.* [6] proposed a method using quaternions for calculating body segment orientations from the angular velocity data of a body-mounted gyroscope; however, the proposed method uses the cyclic properties of gait to compensate for the drift. Another approach using quaternions is proposed in [7]: the initial orientations of the sensor units were estimated using acceleration data during an upright standing position, and the angular displacements were estimated using angular velocity data. An algorithm based on quaternion calculation was implemented for the orientation estimation of the sensor units, which were converted afterwards to the orientation of the body segments by a rotation matrix obtained from a calibration trail. Tong *et al.* [8] derived segment inclinations and the knee angle from segment angular velocities and applied a low-cut high-pass filter on the shank and thigh inclination angle signals, but removing low-frequency information. Favre *et al.* used acceleration data to compensate for the drift in the angular velocity data, but this compensation algorithm needs the dynamical acceleration to be negligible with respect to the gravitational acceleration [9].

Another method to estimate joint angles from measured accelerations is the estimation of angles between sensors and the vertical direction. These methods could became less accurate if the segment accelerations are as large as the gravity [10,11].

The accurate estimation of joint angles and the beginning of the stance phase allows the detection of the gait phases. Indeed, a normal walking gait cycle is divided into eight different gait phases, including initial contact, loading response, mid-stance, terminal stance, pre-swing, initial swing, mid-swing and terminal swing, and each phase is determined by the joint angles and gyros measurement [12].

A different approach was presented in [12], where each single gait phase was detected by means of gyroscopes and accelerometers; the former measured the angular velocity of each segment; the latter measured the inclination of the leg segment in every single gait cycle for periodic recalibration. An estimation algorithm was proposed to estimate segment orientations by integration of the angular velocity obtained from gyroscopes. Furthermore, Willemsen *et al.* [13] estimated joint angles without integration; the method is based on a comparison of signals from accelerometers mounted on adjacent segments of the leg. However, a low-pass filter is requested, therefore introducing a delay.

In many applications, the offset drift is solved by means of the Kalman filter. Cikajlo *et al.* proposed an algorithm where the Kalman filter is used to correct the shank inclination measured by the gyroscope [14]; in addition, the extended Kalman filter [15] and Gaussian particle filter [16] were also used to evaluate the hip angle in a walking cycle from the measurements of the wearable sensors, thus improving accuracy. Furthermore, a neural network [17] was applied for the estimation of ankle, knee and hip joint angles, obtaining good accuracy; however, this method needs training for individual settings before measurements in order to estimate with good accuracy.

Popovic *et al.* [18] proposed a new method for the estimation of absolute segment and joint angles during the gait; absolute angles of each segment were determined by band pass filtering the difference between signals from two accelerometers, while joint angles were evaluated by subtracting absolute angles of the neighboring segment. The offset drift was minimized with a Butterworth filter, and it can be additionally reduced if a high-pass filter is used in conjunction with the low-pass filter. This approach is similar to the method based on multi-rate complementary filtering theory developed in [19] for a navigation system. However, the method encounters an accuracy problem if the gait is slow; therefore, it is not acceptable for patients with a high level of disability. Another method of gait parameters recognition in real-time is proposed in [20], where the wearable system consists of a tri-axial accelerometer, and an autocorrelation procedure estimates the repeating characteristics over the gait periodic signal.

An interesting method was proposed by Watanabe *et al.* [21–23], where signals from the sensor attached on the foot were used in the gait length estimation, and joint angles were calculated as the integral of the difference between angular velocities measured from two gyroscopes attached on adjacent segments. The outputs of accelerometers are filtered with a Butterworth low-pass filter and used to measure inclination, and the offset drift problem was solved by the Kalman filter, used to estimate the error of the joint angle measured by gyroscopes from the differences between angles obtained by the gyroscopes and those by accelerometers.

A strategy to estimate hip and knee joint angles has been recently proposed in [24]. The proposed system is based only on accelerometers and gyroscopes measurements, and an exact solution of a second order kinematic equation gives the estimation of the hip and knee angles. In particular, the algorithm proposed in [24] was tested under walking on a short-distance pathway. Experiments showed a good performance of this algorithm, save for the time interval right after the contact of the foot with the ground, where there are vibrations.

In order to solve these issues in this paper, two methods are proposed to estimate gait parameters based on the complementary filter method [19]: the former merges the method proposed in [24] with the complementary filter method, whereas the latter merges the complementary filter method with the integrated gyro signals. The proposed algorithms estimate hip and knee angles on level ground walking tasks and the transition between the swing and stance phases, *i.e.*, the instant of contact of the foot with the ground.

In the Section 2, we provide the methods for the hip and knee angles estimation and the complementary filter method used for the results comparison, whereas in Section 3, we present the sensor system. Furthermore, some experimental findings are shown in Section 4, and Section 5 deals with some conclusions.

## 2. Methods

### 2.1. Acceleration Propagation-Based Method (APB)

**Figure 1.**
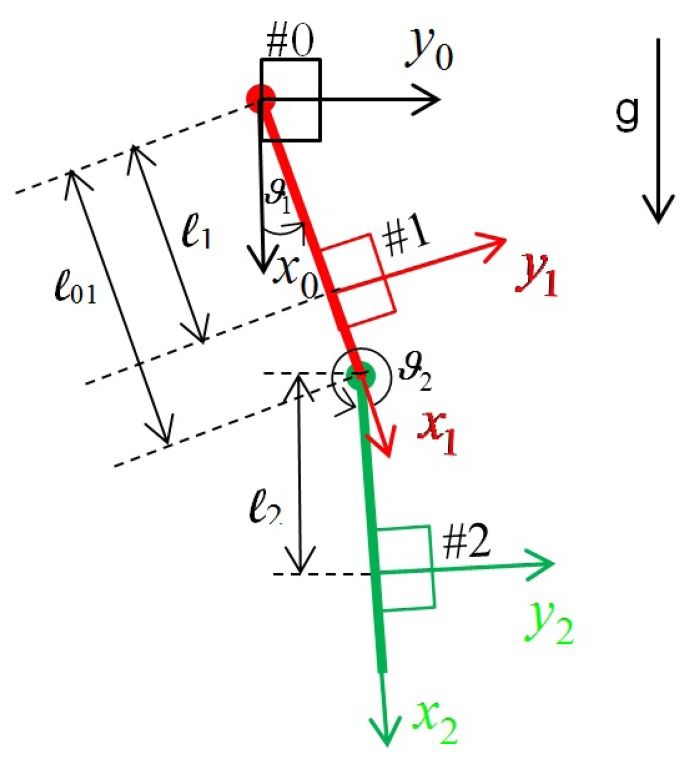
Kinematic scheme of a leg.

In this section, the acceleration propagation-based method originally proposed in [24] will be briefly reviewed. The sensor system is based only on low-cost inertial sensors, such as accelerometers and gyroscopes. The kinematic scheme, for each leg, is shown in Figure 1. In the same Figure, the location of the sensors is shown. Each group consist of one accelerometer and one gyro. A corresponding frame is located for each group of sensors and represents the position and orientation of the sensors.

A significant problem for the measurement of joint angles with gyroscopes is the error accumulation in the integral value, due to the pronounced drift. In order to avoid this drawback, the following second order kinematic model between two consecutive leg segment can be considered.

First, let us consider the kinematic relation expressing the position of the knee accelerometer, *P*_1_, with reference to that of the hip, *P*_0_:

(1) $$P_{0}=P_{1}+R_{1}^{0}P_{1,0}^{1}=P_{1}-l_{1}x_{1}$$

where:

(2) $$P_{1,0}^{1}=\left[\begin{array}{ccc} -l_{1}, & 0, & 0 \end{array}\right]$$

*l*_1_ is the position of the knee accelerometer (with respect to the coordinate Frame 0), and the rotation matrix is defined as $R_{1}^{0}=(\begin{array}{ccc} x_{1} & y_{1} & z_{1} \end{array})$, with *x*_1_, *y*_1_, *z*_1_, the direction vectors. Indeed,

(3) $$x_{1}=\left[\begin{array}{ccc} c_{1}, & s_{1}, & 0 \end{array}\right]\;,y_{1}=\left[\begin{array}{ccc} -s_{1}, & c_{1}, & 0 \end{array}\right]\;,z_{1}=\left[\begin{array}{ccc} 0, & 0, & 1 \end{array}\right]$$

and:

(4) $${ẋ}_{1}={\dot{\theta }}_{1}y_{1}$$

(5) $${ẏ}_{1}=-{\dot{\theta }}_{1}x_{1}$$

The second order kinematic model can be obtained from Equation (1), making the derivative:

(6) $${Ṗ}_{0}={Ṗ}_{1}-l_{1}{\dot{\theta }}_{1}y_{1}$$

(7) $${\ddot{P}}_{0}+l_{1}{\ddot{\theta }}_{1}y_{1}-l_{1}{\dot{\theta }}_{1}^{2}x_{1}={\ddot{P}}_{1}$$

where *θ̇*_1_ is the measure from the knee gyroscope.

As the measure from the *i* – *th* accelerometer can be expressed as:

(8) $${\ddot{P}}_{i,m}={\ddot{P}}_{i}+gx_{0}$$

with *g* the gravitational acceleration, therefore, the second order kinematic relation (7), by adding *gx*_0_ on both sides, becomes:

(9) $${\ddot{P}}_{0,m}+l_{1}{\ddot{\theta }}_{1}y_{1}-l_{1}{\dot{\theta }}_{1}^{2}x_{1}={\ddot{P}}_{1,m}$$

which can be equivalently written as:

(10) $${\ddot{P}}_{0,mx}x_{0}+{\ddot{P}}_{0,my}y_{0}={\ddot{P}}_{1,mx}^{1}x_{1}+{\ddot{P}}_{1,my}^{1}y_{1}+l_{1}{\dot{\theta }}_{1}^{2}x_{1}-l_{1}{\ddot{\theta }}_{1}y_{1}$$

where *P̈*_0,*mx*_, *P̈*_0,*my*_ and ${\ddot{P}}_{1,mx}^{1}$, ${\ddot{P}}_{1,my}^{1}$ are measurements from Accelerometers 0 and 1, respectively.

Multiplying Equation (10) by $x_{1}^{T}$ and $y_{1}^{T}$, it can be obtained, respectively:

(11) $$\begin{array}{c} {\ddot{P}}_{0,mx}c_{1}+{\ddot{P}}_{0,my}s_{1}-l_{1}{\dot{\theta }}_{1}^{2}={\ddot{P}}_{1,mx}^{1}\\ \begin{array}{c} -{\ddot{P}}_{0,mx}s_{1}+{\ddot{P}}_{0,my}c_{1}+l_{1}{\ddot{\theta }}_{1}={\ddot{P}}_{1,my}^{1} \end{array} \end{array}$$

which can be solved for *c*_1_ and *s*_1_. Then, obtaining the hip angle is straightforward.

In the same way for the second link, the position between the hip and ankle accelerometer is expressed by:

(12) $$P_{0}+l_{01}x_{1}+l_{2}x_{2}=P_{2}$$

where *l*_01_ is the position of the origin, *O*_1_ (with respect to coordinate Frame 0), *l_2_* is the position of the ankle accelerometer (with respect to the coordinate Frame 1), and the rotation matrix is defined as $R_{2}^{0}=(\begin{array}{ccc} x_{2} & y_{2} & z_{2} \end{array}).$

The two successive time differentiation of Equation (12) are:

(13) $${Ṗ}_{0}+l_{01}{\dot{\theta }}_{1}y_{1}+l_{2}({\dot{\theta }}_{1}+{\dot{\theta }}_{2})y_{2}={Ṗ}_{2}$$

(14) $${\ddot{P}}_{0}-l_{01}{\dot{\theta }}_{1}^{2}x_{1}-l_{2}{\left({\dot{\theta }}_{1}+{\dot{\theta }}_{2}\right)}^{2}x_{2}+l_{01}{\ddot{\theta }}_{1}y_{1}+l_{2}({\ddot{\theta }}_{1}+{\ddot{\theta }}_{2})y_{2}={\ddot{P}}_{2}$$

where (*θ̇*_1_ + *θ̇_2_*) can be measured from the ankle gyroscope. Therefore, for the second link, the second order kinematic relation is:

(15) $${\ddot{P}}_{0,mx}x_{0}+{\ddot{P}}_{0,my}y_{0}-l_{01}{\dot{\theta }}_{1}^{2}x_{1}-l_{2}{\left({\dot{\theta }}_{1}+{\dot{\theta }}_{2}\right)}^{2}x_{2}+l_{01}{\ddot{\theta }}_{1}y_{1}+l_{2}({\ddot{\theta }}_{1}+{\ddot{\theta }}_{2})y_{2}={\ddot{P}}_{2,mx}^{2}x_{2}+{\ddot{P}}_{2,my}^{2}y_{2}$$

where ${\ddot{P}}_{2,mx}^{2}$ *and* ${\ddot{P}}_{2,my}^{2}$ are the measures from Accelerometer 2 (ankle).

As in the case of the first link, multiplying Equation (15) by $x_{2}^{T}$ and $y_{2}^{T}$, it can be obtained, respectively:

(16) $$\begin{array}{c} {\ddot{P}}_{0,mx}c_{12}+{\ddot{P}}_{0,my}s_{12}-l_{01}{\dot{\theta }}_{1}^{2}c_{2}-l_{2}{\left({\dot{\theta }}_{1}+{\dot{\theta }}_{2}\right)}^{2}+l_{01}{\ddot{\theta }}_{1}s_{2}={\ddot{P}}_{2,mx}^{2}\\ -{\ddot{P}}_{0,mx}s_{12}+{\ddot{P}}_{0,my}c_{12}+l_{01}{\dot{\theta }}_{1}^{2}s_{2}+l_{01}{\ddot{\theta }}_{1}c_{2}+l_{2}({\ddot{\theta }}_{1}+{\ddot{\theta }}_{2})={\ddot{P}}_{2,my}^{2} \end{array}$$

where *c*_12_ and *s*_12_ are the cosine and the sine of the angle (*θ*_1_ + *θ*_2_). With some algebraic manipolation, Equation (16) become:

(17) $$\begin{array}{c} {}[{\ddot{P}}_{0,mx}c_{1}+{\ddot{P}}_{0,my}s_{1}-l_{01}{\dot{\theta }}_{1}^{2}]c_{2}+[{\ddot{P}}_{0,my}c_{1}-{\ddot{P}}_{0,mx}s_{1}+l_{01}{\ddot{\theta }}_{1}]s_{2}={\ddot{P}}_{2,mx}^{2}+l_{2}{\left({\dot{\theta }}_{1}+{\dot{\theta }}_{2}\right)}^{2}\\ \left[{\ddot{P}}_{0,my}c_{1}-{\ddot{P}}_{0,mx}s_{1}+l_{01}{\ddot{\theta }}_{1}]c_{2}-[{\ddot{P}}_{0,mx}c_{1}+{\ddot{P}}_{0,my}s_{1}-l_{01}{\dot{\theta }}_{1}^{2}]s_{2}={\ddot{P}}_{2,my}^{2}-l_{2}({\ddot{\theta }}_{1}+{\ddot{\theta }}_{2}\right) \end{array}$$

where *c_2_* and *s_2_* are the cosine and the sine of the knee angle.

Considering the kinematic relation (11), Equation (17) becomes:

(18) $$\begin{array}{c} {}[{\ddot{P}}_{1,mx}^{1}+(l_{1}-l_{01}){\dot{\theta }}_{1}^{2}]c_{2}+[{\ddot{P}}_{1,my}^{1}-(l_{1}-l_{01}){\ddot{\theta }}_{1}]s_{2}={\ddot{P}}_{2,mx}^{2}+l_{2}{\left({\dot{\theta }}_{1}+{\dot{\theta }}_{2}\right)}^{2}\\ \left[{\ddot{P}}_{1,my}^{1}-(l_{1}-l_{01}){\ddot{\theta }}_{1}]c_{2}-[{\ddot{P}}_{1,mx}^{1}+(l_{1}-l_{01}){\dot{\theta }}_{1}^{2}]s_{2}={\ddot{P}}_{2,my}^{2}-l_{2}({\ddot{\theta }}_{1}+{\ddot{\theta }}_{2}\right) \end{array}$$

which can be solved for *c*_2_ and *s*_2_, therefore allowing one to estimate the knee angle, *θ*_2_.

The kinematic relations (18) are analogous to the Relations (11), except for the compensation terms, which depends on the difference *(l*_1_ — *l*_01_), namely the distance between the knee accelerometer and the origin of Frame 1. Therefore, if the knee accelerometer is put as *l*_1_ = *l*_01_, the system will become more robust, and the joint angle computation is a simple propagation of the same formula.

Note that a similar approach is proposed in [25], where the quaternion describing the orientation of the accelerometer is computed by the means of an optimization algorithm, but assuming the dynamic acceleration to be negligible compared to the static acceleration due to gravity.

### 2.2. Complementary Filter Method (CF)

The complementary filter is one of the most powerful and simple methods for the data fusion of different sources of information, which are complementary in a frequency domain [19]. Being a standard *de facto*, the CF approach will be compared to the acceleration propagation-based method (APB) on the angle estimation under walking on the short-distance pathway. Moreover, the others method proposed in this work are based on the complementary filter method (CF) and will be compared to it. For this reason, in the following, a brief introduction to the CF method will be given.

For every signal, *P*(*s*), and transfer function *W*(*s*), it is possible to write:

(19) P(s)W(s)+P(s)(1−w(s))=P(s)

If *W*(*s*) has low-pass characteristics, 1 – *W*(*s*) has complementary high pass characteristics; then it is possible to obtain an approximation of the signal, *P*, by filtering two complementary sources of information:

(20) $$P_{\text{low}}W_{PB}+P_{\text{high}}(1-W_{PB})=P$$

where *P_low_* and *P_high_* are a low frequency and high frequency approximation of *P*, respectively.

In many applications, the high frequency approximation of the signal can be obtained by the integration of a velocity signal, which shows a low frequency drift phenomena. Then, Equation (20) can be written as:

(21) $$P_{\text{low}}W_{PB}+\frac{\upsilon_{\text{high}}}{s}(1-W_{PB})=P$$

The most simple implementation of the CF can be obtained starting from the low-pass filter:

(22) $$W_{PB}=\frac{1}{\tau_{p}s+1}$$

which implies that Equation (20) can be implemented as:

(23) $$(P_{\text{low}}+\tau_{p}\upsilon_{\text{high}})W_{PB}=P$$

In this application, the estimation of the position is based on measurements provided by accelerometers (*P_low_*) and gyroscopes (*υ_high_*). Indeed, accelerometers provide accurate information at a low frequency; however, gyroscopes show drift phenomena in the same frequency band; therefore, they are useful at higher frequencies.

### 2.3. Mixed Complementary Filter-APB (CF-APB)

The classical implementation of the CF requires the use of the accelerometer as an inclinometer, *i.e.*, the dynamical acceleration must be negligible with respect to the gravitational one. Unfortunately, this hypotheses is also not true at low frequencies. For example, if the angle velocity is constant, a constant centrifugal acceleration disturbance will affect the radial component of the acceleration.

To solve this problem, a new complementary filter method has been introduced:

(24) $$P_{\text{APB}}W_{PB}+\frac{\upsilon_{\text{gyro}}}{s}(1-W_{PB})=P$$

consisting of the APB method for the low frequency approximation, *P_APB_* and the gyro velocity for high frequency, *υ_gyro_*.

### 2.4. Intelligent Complementary Filter (ICF)

The APB algorithm, as well as the complementary filter method, as is shown in the next section, have an accurate performance in the walking tests, where there are not considerable fast variations of joint angles nor contacts of the foot with the ground, the latter bringing to oscillations of the position estimation for a short time interval due to the second order characteristic of the accelerometer. The proposed intelligent complementary filter (ICF) tries to solve this problem by using complementary filters with a rate limiter and the integrated signal from gyros blended together.

The general idea of the method proposed in this section is based on the fact that during oscillations, due to the contact, the integrated gyroscope signal is accurate enough. Then, the accelerometer signal, bringing oscillations in the time interval right after the contact of the foot with the ground, should not be considered in this circumstance, and the integrated gyroscope signal will be considered instead.

**Figure 2.**
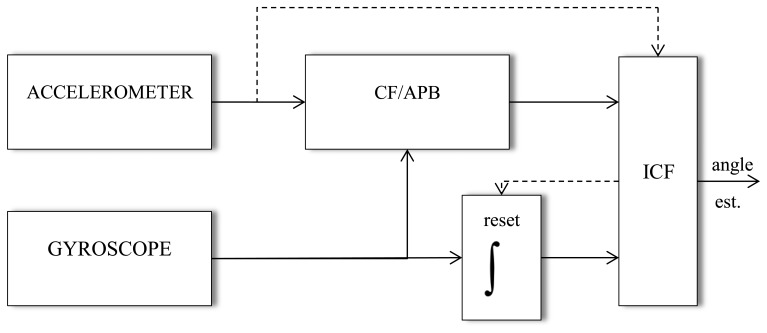
Schematic structure of the intelligent complementary filter (ICF) method.

The scheme of the ICF estimation method is shown in the Figure 2. With reference to this Figure, the estimates of the joint angles is normally obtained by means of the CF or APB method. When the rate of estimation exceeds a given maximum value, the integrated gyroscopic signal is used instead. Finally, when the gap is over, the joint angles could be estimated again by means of the CF or APB. Other criteria could be considered in order to detect contact and switch, consequently, between methods. However, the proposed one gives the best experimental result. The value of the rate limiter is chosen as equal to the maximum value of the gyro sensor.

## 3. Sensor System

**Figure 3.**
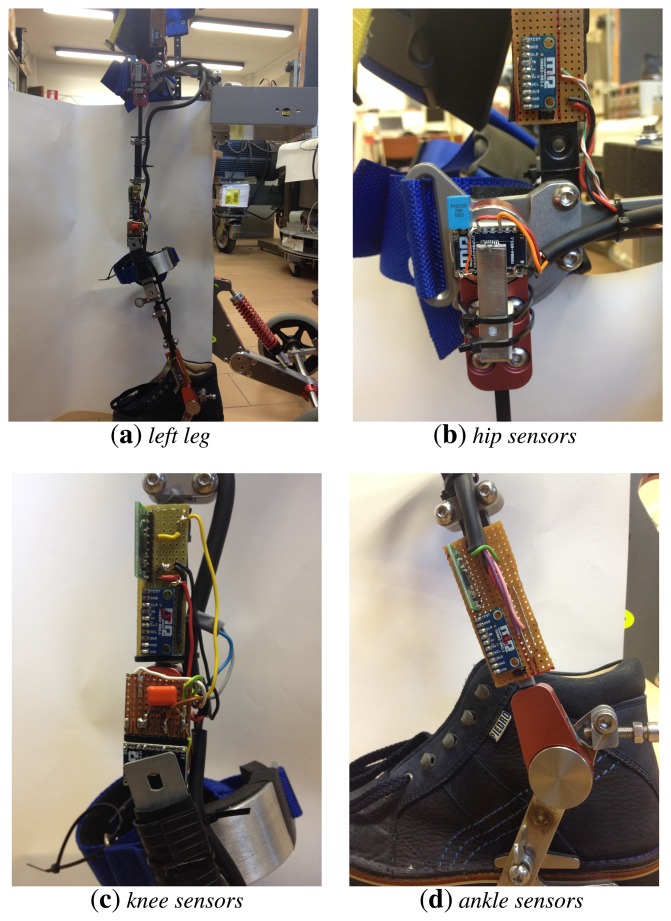
(**a**) Left leg of the modified commercial device for assisted gait (NF-Walker) used in the experiments; (**b**) particulars of the hip accelerometer and encoder; (**c**) particulars of the knee gyro, accelerometer and encoder; (**d**) particulars of the ankle gyro and accelerometer.

The sensor system consists of accelerometers and gyroscopes put near the knee and the ankle (see Figure 3); magnetic encoders were also put on the hip and knee joints in order to compare the results of the estimation algorithms to a reference angular measurement.

The experimental equipment is comprised of:

- LPY510Al (ST), an analog, low-power dual-axis micro-machined gyroscope, capable of measuring the angular rate along pitch and yaw axes. It provides excellent temperature stability and high resolution. The gyroscope allows band limiting of the output rate response through the integrated low-pass filter.
- MMA7361L (FreeScale), a capacitive low power, three-axis accelerometer, integrating a voltage regulator and a low-pass filter.
- ARDUINO DUE, a microcontroller board based on the Atmel SAM3X8E ARM Cortex-M3 CPU, providing 54 digital input/output pins, 12 analog inputs (12 bits of resolution), an 84-MHz clock, 96 KBytes of SRAM, 512 KBytes of Flash memory for code.
- AS5045 contactless magnetic rotary sensor for accurate angular measurement over a full turn. It is a system-on-chip, combining integrated hall elements, an analog front end and digital signal processing in a single device.

## 4. Results and Discussion

The proposed algorithm has been tested on angle measurements of the hip and knee of a commercial device for assisted gait: the NF-Walker [26]. The sensors were attached on the leg, as shown in the previous section.

The experiments performed have the main purpose of comparing the different ways of estimating hip and knee angles with direct angular measurement from encoders.

In addition, these algorithms are evaluated on an over-grounded walking trial. However, due to the limited laboratory size (therefore, the limited walking distance), this kind of measurement was restricted to only a few steps.

### 4.1. Experiment 1: Evaluation of the CF-APB Method

This experiment is performed to evaluate the performance of the mixed complementary filter-APB (CF-APB) algorithm on an over-ground walking trial.

**Figure 4.**
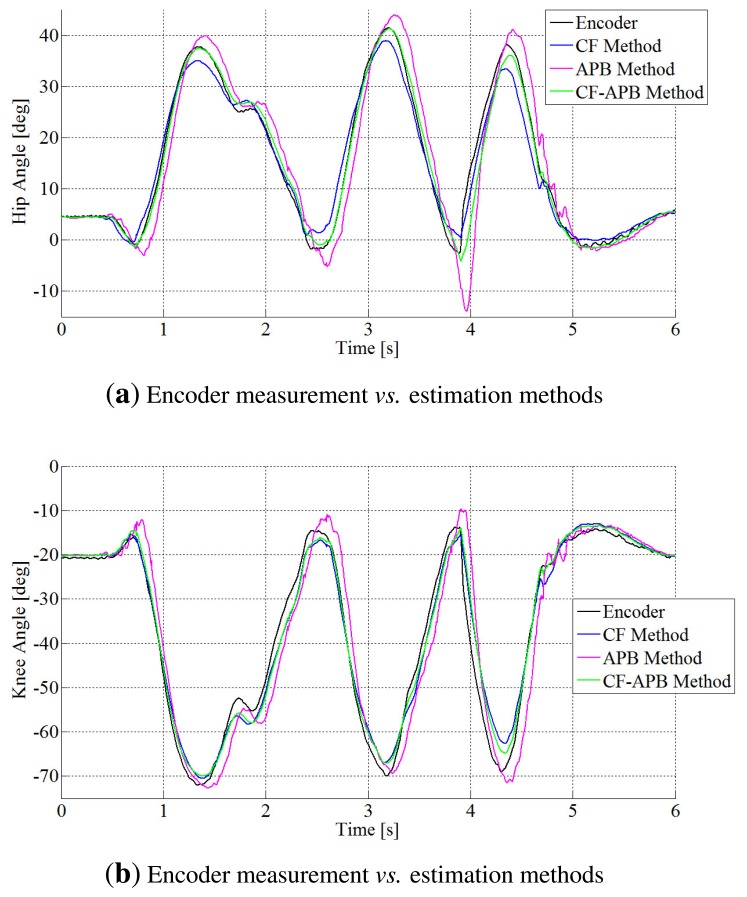
(**a**) Comparison between acceleration propagation-based method (APB), complementary filter (CF) and CF-APB performances vs. encoder measurement for hip angle estimation. (**b**) Comparison between APB, CF and CF-APB performances vs. encoder measurement for knee angle estimation.

In the Figure 4, a comparison of the performances of the APB method with the classic CF method is shown, and the mixed complementary filtering approach (CF-APB), as well.

From this figure, it is pointed out that the APB algorithm, as well as the complementary filter method show good accuracy for the angle estimation, save for a time interval after the contact, where there are vibrations.

**Figure 5.**
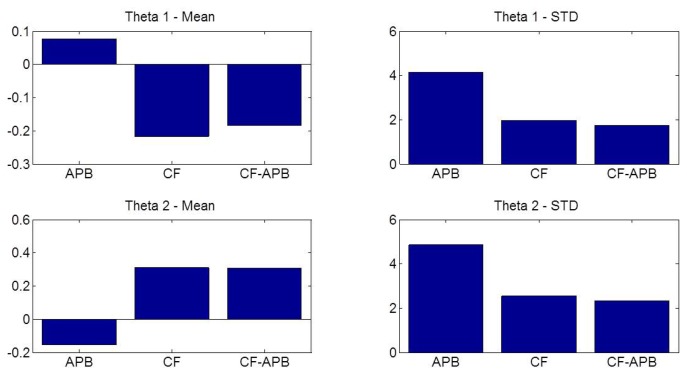
Statistic comparison between APB, CF and both bound together (CF-APB).

Finally, in Figure 5, it is pointed out, by means of a comparison between means and standard deviations, the improved performance of the CF-APB method for both hip and knee angle estimations. As we can see, comparing the APB and CF methods, the former presents a better value of means, whereas the latter produces a better value of the standard deviation. Therefore, the CF-APB algorithm, that is, both the previous algorithms bound together, presents a mixing of the advantages of both methods.

### 4.2. Experiment 2: Evaluation of the ICF Method

This experiment is performed to evaluate the performance of the intelligent complementary filter (ICF) algorithm on an over-ground walking trial.

**Figure 6.**
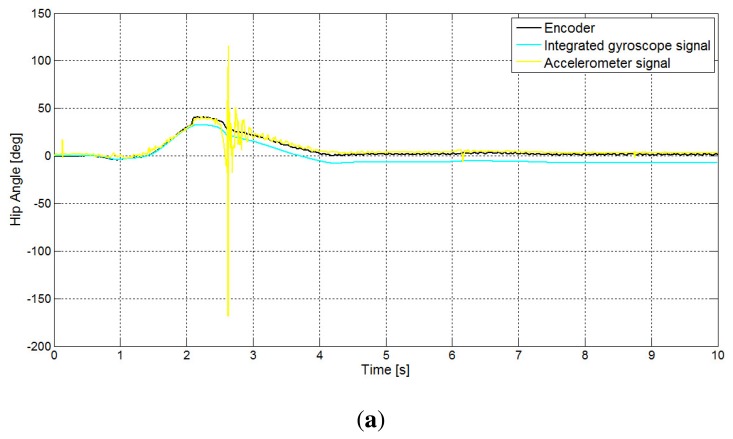

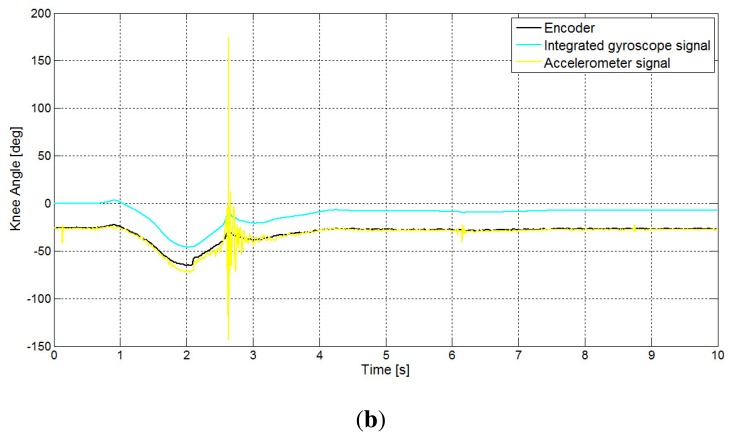
The integrated gyroscope signal and the accelerometer-based position estimation: (**a**) hip angle; (**b**) knee angle.

As already said, both the CF and ICF methods get information from a position signal obtained from the accelerometer and a second piece of position information obtained from the integral of the gyros. In Figure 6, these two signals are shown, with reference to the hip and knee angles. As is shown in the figure, the accelerometer-based position estimation signal fails to track the true position when contact of the foot with the ground occurs. On the other side, from the same signal, it is possible to easily detect the beginning of the stance phase, which is the instant where the foot comes into contact with the ground. The integral of the gyroscope, however, is not affected by the contact, but is affected by drift.

**Figure 7.**
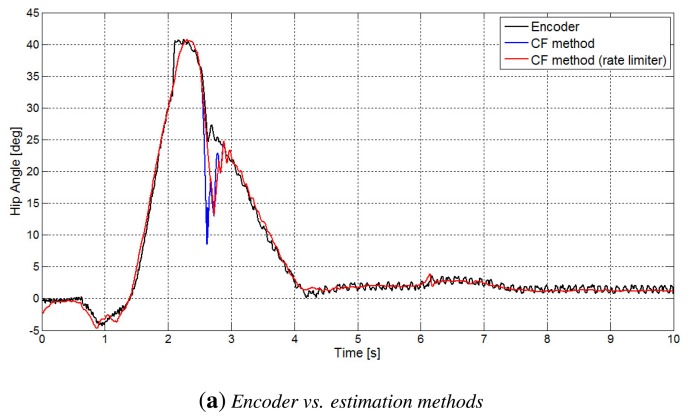

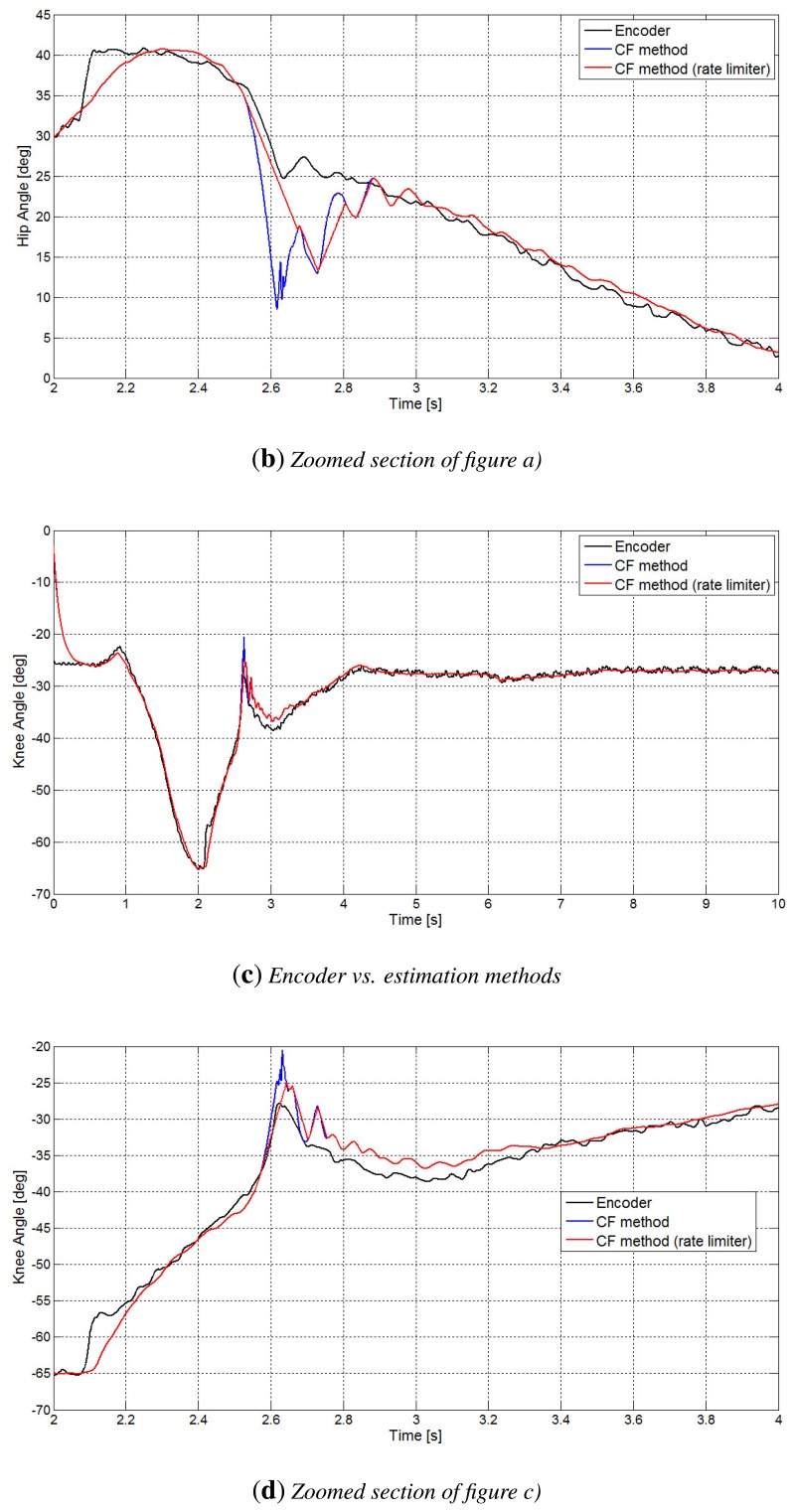
Estimation of the angles by means of the CF method vs. encoder measurement: (**a**) hip angle; (**b**) particulars of the hip angle estimation; (**c**) knee angle; (**d**) particulars of the knee angle estimation.

In the Figure 7, the behavior of the CF method for both hip and knee angles is shown. In particular, in Figure 7b–d is pointed out the occurrence of a consistent estimation error during the contact.

**Figure 8.**
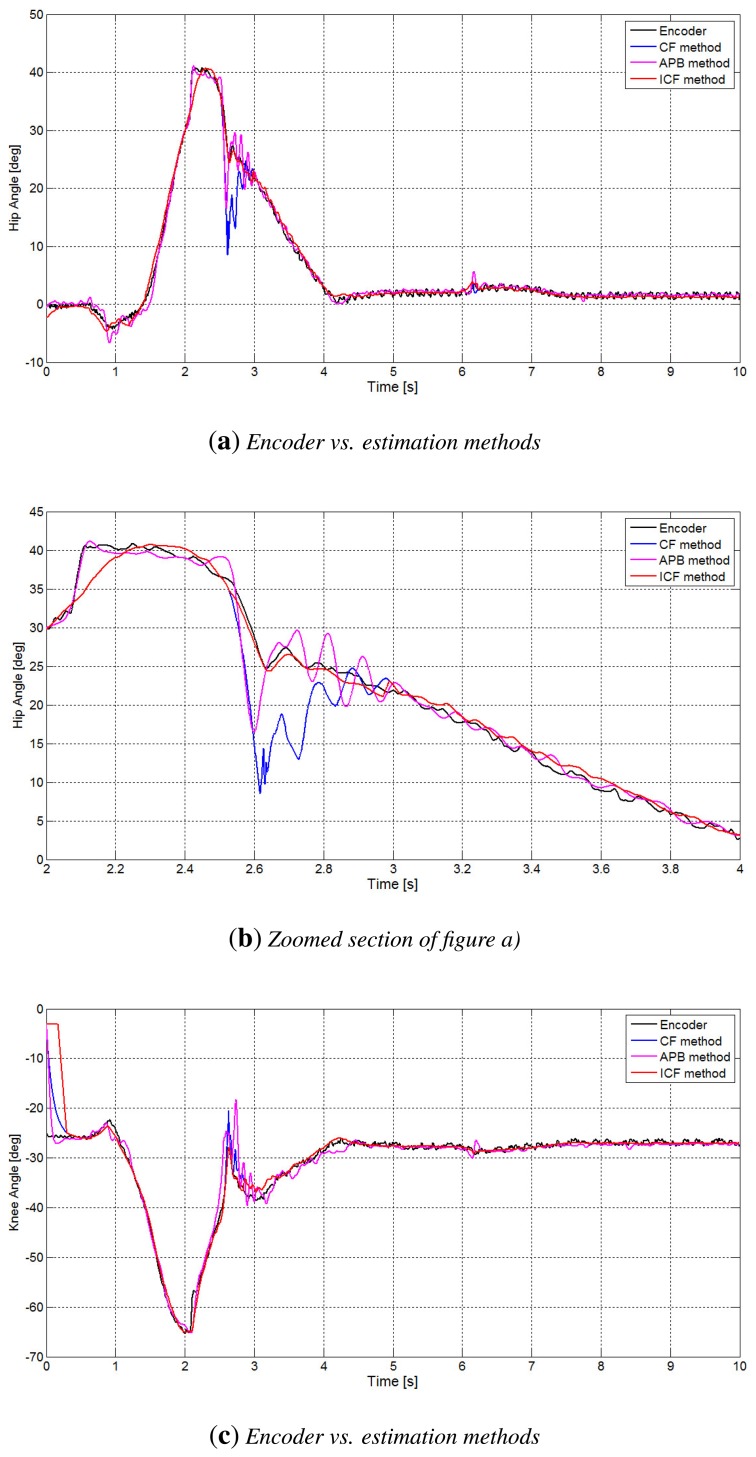

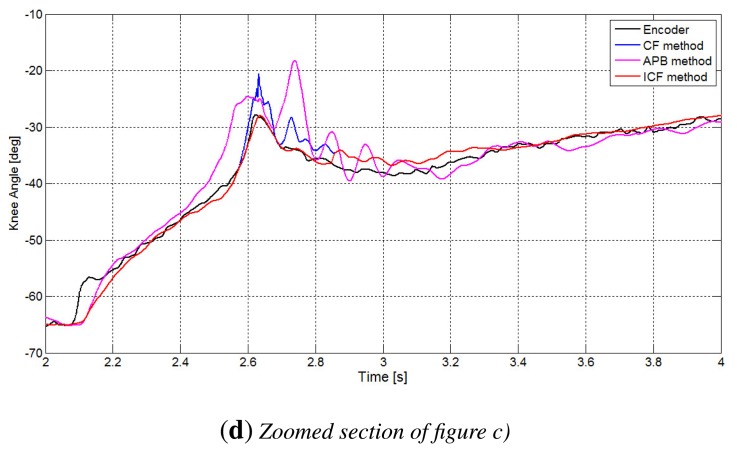
Comparison between the APB, CF and intelligent complementary filter (ICF) methods vs. encoder measurement: (**a**) hip angle estimation; (**b**) particulars of the comparison between estimation methods for the hip angle; (**c**) knee angle estimation; (**d**) particulars of the comparison between estimation methods for the knee angle.

Finally, in Figure 8, there is a comparison of the performances of the APB, CF and ICF methods. In particular, in Figure 8b–d, the improvement of the performance of the ICF method is evident, especially in the time interval concerning the contact issues, where both the APB algorithm and the CF method present oscillations, and the estimation is not really accurate.

## 5. Conclusions

In this paper, a low-cost sensor system, based on accelerometer and gyroscope measurements, has been studied in order to estimate hip and knee angles during the rehabilitation and monitoring of patients.

The main purpose of the work is to avoid the significant problem of the error accumulation due to the offset drift caused by gyroscopes and the oscillations related to the time interval right after the contact of the foot with the ground, caused by the accelerometer.

In order to solve these problems, some algorithms have been proposed and experimentally evaluated and compared, estimating hip and knee angles during gait. In particular, as we can see from the experimental results, the acceleration propagation-based method (APB) and the complementary filter (CF) method have good performance, save for the time interval where the contact of the foot with the ground occurred. These problems were solved with the mixed complementary filter-ABP method (CF-APB) and the intelligent complementary filter method (ICF).

In addition, it is possible to use the trigger signals of accelerometers in order to detect the beginning of the stance phase, namely the initial contact of the foot with the ground.

The estimated gait parameters could be used in order to detect accurately gait phases, as is shown in [12]. The proposed algorithms have been tested on the angle measurement of the hip and knee of a commercial device for assisted gait, the NF-Walker.

Future developments of this algorithm will be the development of a mechanistic system for the walking assistance of motor-impaired child. In particular, hip and knee angle estimations, with both angular velocity and acceleration measurements and the detection of the beginning of stance phase will be shortly used to detect gait phase in a totally wearable system and, afterwards, to develop a proper control law to activate pneumatic muscles, put on the ortheses, in order to support the rehabilitation gait patterns.
